# Estimating the time-varying generation rate of acetic acid from an all-purpose floor cleaner

**DOI:** 10.1038/s41370-019-0142-5

**Published:** 2019-05-14

**Authors:** Susan Arnold, Gurumurthy Ramachandran, Hannah Kaup, Joseph Servadio

**Affiliations:** 1 Division of Environmental Health Sciences, School of Public Health, University of Minnesota, MMC 807, Room 1239, 420 Delaware Street SE, Minneapolis, MN 55455 USA; 2 Department of Environmental Health and Engineering, Bloomberg School of Public Health, Johns Hopkins University, Baltimore, MD 21205 USA

**Keywords:** generation rate, acetic acid mixture, evaporation rate, exposure model

## Abstract

Understanding the relationship between consumer product use and risk of adverse health outcomes facilitates appropriate risk management and product stewardship. A preferred method for estimating the systemic and respiratory tract exposure and dose tailored to cleaning products use has been proposed, refining previously issued exposure guidance. Consistent with other exposure and risk-assessment frameworks, it is dependent upon high-quality exposure determinant data that also serve as model inputs. However, as publicly available exposure determinant data are scarce, the risk assessor is left with the option of estimating determinants such as the generation rate or employing empirical methods to estimate them. When the exposure scenario involves a chemical mixture, estimating the generation rate may not be feasible. We present an approach for estimating the time-varying generation rate of an aqueous acetic acid mixture representative of the base formulation for many consumer and DIY cleaning products that was previously assessed in a screening-level assessment. The approach involved measuring the evaporation rate for a reasonable worst-case scenario under controlled conditions. Knowing the mass applied, a time-varying generation rate was estimated. To evaluate its portability, a field study was conducted in a home where measurements were collected in an all-purpose room with the exterior door open (Room 1) and closed (Room 2), and a bathroom (Room 3) using a portable Fourier Transform Infrared (FTIR) spectrophotometer. Acetic acid concentrations were modeled using two common indoor air models, the Well Mixed Room model. Measured and modeled acetic acid concentrations were compared, with the WMR 95% confidence intervals encompassing measured concentrations for all three rooms, supporting the utility of the approach used and portability of the generation rate derived from it.

## Introduction

Safety assessments are important tools in understanding the relationship between consumer product use and risk of adverse health outcomes. Safety assessments include both estimation of dose–response and estimation of exposure to determine the Margin of Safety (MOS), defined as the ratio of the inhalation guidance value, provisional safe dose, or Toxicological Threshold of Concern to the human exposure estimate [1]. Although consumer product use can lead to both dermal and inhalation exposure, the focus of this paper is inhalation exposure. Assessing exposures requires characterizing specific exposure determinants in each scenario, as the frequency, duration, route, and mode of use or application affect the emission and, therefore, the delivered dose. Exposures may be generated by a single product containing a single chemical, a single product containing multiple chemicals, a single chemical found in multiple products, or multiple chemicals found in multiple products. Product use and application also influence the way in which the dose is delivered and therefore must also be understood [2]. Cleaning products, for instance, may be applied by spraying, wiping, or pouring. Chemicals from the cleaning products may become airborne in the form of droplets when the product is sprayed or vaporize into the room air, exposing the user inhaling the contaminated air. Several studies suggest an association between cleaning activities, cleaning products, and asthma [3–8].

A preferred method for estimating the systemic and respiratory tract exposure and dose tailored to cleaning products use has been proposed, refining previously issued exposure guidance [1]. These refinements suggest a tiered assessment framework that provides a MOS estimate for cleaning product use and potential asthma responses. In all cases, robust dose–response data are required. When high-quality model inputs are not available, plausible worst-case estimates generated from screening level values can be used [9]. These highly conservative inputs may result in exposure estimates that significantly overestimate real-world exposures. In situations where these worst-case estimates exceed the MOS, refinement of the model inputs may be necessary so that a more realistic exposure estimate can be developed. Tier II assessments use vetted model estimates to generate human inhalation exposure estimates that are based on physiochemical model inputs sourced from the publicly available and peer-reviewed literature. From these models, reasonably accurate exposure estimates can be generated. Top-tier assessment, denoted Tier III here, aligns with the EPA, AIHA [11], and SCSS [9] exposure assessment and modeling frameworks. In all cases, top-tier assessments require robust hazard and exposure data.

Finding high-quality publicly available model inputs for Tier II assessments remains a challenge, as these data are scarce. High-quality generation rate data are especially difficult to find in the literature. A mass balance approach, accounting for the mass of the chemical used, can provide a conservative estimate of the generation rate, supporting a screening-level exposure assessment. Physical and chemical principles can be applied to estimate generation rates (G) of products that contain pure or relatively pure volatile and semi-volatile chemicals. In liquids conforming to the ideal gas laws, Raoult’s Law or Henry’s Law can be useful in estimating the vapor pressure of a chemical constituent of a mixture, and thus gain a general understanding of how much and how fast that chemical will evaporate from the mixture. However, emission rates derived from these laws become less reliable, as that mixture departs from ideal behavior. For products applied by spraying or dusty products emitting aerosols, estimating generation rates based on chemical or physical principles is challenging at best; in many cases, they must be estimated using empirical approaches [10, 11].

To support the development of refined exposure assessments for cleaning products containing acetic acid, a reasonably accurate generation rate is needed. As the generation rate is independent of room size, air exchange rate, and exposure duration, determining this model parameter for a common scenario under conditions that are representative of many use scenarios will yield a generation rate that is portable, i.e., applicable to other similar scenarios. Specifically, in this case, it would support a Tier II assessment of a multitude of surface-cleaning scenarios for products formulated from aqueous mixtures of acetic acid. Thus, this research presents an approach for estimating and evaluating the time-varying generation rate, *G*_n_(*t*) for acetic acid evaporating from an all-purpose cleaner, and assesses its use in estimating exposure in a field environment using the Well Mixed Room (WRM) model, which is frequently used to model indoor airborne contaminant concentrations.

## Methods

A two-part study was designed to estimate the evaporation rate *k* and time-varying generation rate *G*_n_(*t*) under a set of conditions deemed representative of a surface-cleaning scenario. First, tests were conducted in a full-size exposure chamber [10] to estimate *k* These tests were necessary, because *k* is scenario specific, meaning it will vary based on a number of factors such as temperature of the liquid and air temperature above it, humidity, airflow over the pool, and thickness and surface area of the liquid pool. The chamber study allows us to keep these factors constant, changing only the airflow rate.

**Fig. 1:**
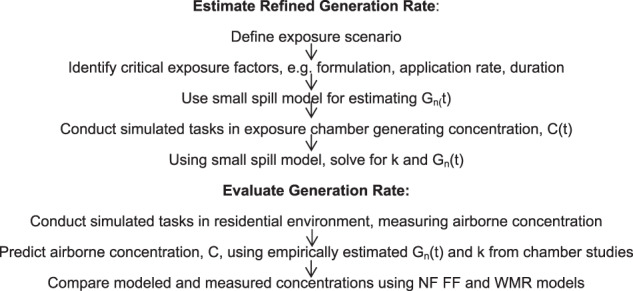
Study process applied to estimate and evaluate a refined *G* from a consumer formulation all-purpose cleaner. Estimating and evaluating a time-varying generation rate followed a two-phased approach. The first phase was conducted in a chamber to estimate a time-varying generation rate *G*_n_(*t*). The second phase occurred in three residential environments, where airborne concentrations were measured and subsequently compared with modeled airborne concentrations based on *G*_n_(*t*)

Knowing the mass applied at each interval, *G*_n_(*t*) for acetic acid, a component of the floor cleaner was estimated. Second, a field study was conducted to evaluate the time-varying generation rate in a residential environment under similar conditions. The approach is outlined in Fig. 1.

Floor-mopping simulations were conducted in a controlled, well-mixed chamber [12]. Product-use conditions believed to represent reasonable worst-case conditions informed the study design. The evaporation rate denoted by *k* (min^−1^) and a time-varying mass emission rate *G*_n_(*t*) (mg/min) were estimated by first measuring the airborne mass concentrations in the chamber. From these measurements, and using a small spill generation rate model [13], a value for *k* and the time-varying mass emission rate *G*_n_(*t*) (mg/min) could be estimated. These values of *k* and *G*_n_(*t*) were subsequently evaluated by first predicting the airborne concentrations in a residential all-purpose room with hardwood floors and separately, in a bathroom with ceramic tile using two models, the WMR model [13]. Finally, the portability of *G*_n_(*t*) was evaluated for its value in predicting exposures beyond the chamber by comparing measured acetic acid concentrations to modeled concentrations.

### Scenario

A representative floor-mopping scenario was defined by and is described in detail in Maier et al. [1]. Briefly, the scenario involves mopping a floor using a generic consumer product formulation for an all-purpose cleaner containing acetic acid. The application rate (mass per unit time) and load (mass per unit area) followed the scenario described in Maier et al. [1]. The formulation used was a 4% aqueous acetic acid mixture, which is the mid-range concentration for acetic acid sold as consumer products. In practice, when used in a Do-It-Yourself cleaning mixture, this concentration is likely further diluted before use as a surface cleaner. Thus, the 4% serves as a reasonable worst-case concentration. Glacial acetic acid (Fisher Scientific, Pittsburgh, PA) was added to distilled water in proportions of ~1 part acetic acid to 24 parts water, by volume. Eucalyptus oil in the amount of 60 drops (Now Essentials, Bloomingdale, IL) was added for fragrance and surface tension properties.

### Chamber study

The simulation was designed to ensure that the volume of floor cleaner and area to which it was delivered was consistent across the duration of the study. The chamber’s impervious stainless steel floor and walls maximized the amount of acetic acid available to evaporate into the room air, supporting a worst-case scenario. The chamber dimensions are 2.83 × 2.1 × 1.98 feet with a total area of 6.01 m^2^. The floor was demarcated into 0.09 m^2^ (1 ft^2^) blocks. The chamber was equipped with two Air King adjustable-height three-speed fans equipped with tilting heads (W.W. Grainger, Inc., www.grainger.com). These were set on the lowest setting to ensure good mixing within the chamber [12]. Using a 10 ml glass pipette (Kimax^TM^, Sigma Aldrich), 10 mL of floor cleaner containing 420 mg acetic acid was delivered to one block, essentially creating a small spill. We then mopped the surface area of that block for 1 min using a Swiffer® mop and disposable pad (Proctor & Gamble, Cincinnati, OH). These steps ensured that factors affecting the evaporation rate, such as the surface length of the liquid pool, surface temperature of the pool, and air speed over the pool discussed above were consistent from “spill” to “spill” and across all simulations. This delivery and mopping process was repeated with each block until the six blocks (0.56 m^2^/6 ft^2^) were mopped. The simulation lasted 6 min, at which time the study personnel exited the chamber, leaving the mop with pad in the room. Airborne acetic acid measurements were collected for an additional 10 min to evaluate post-mopping concentrations. A new pad was used for each test.

Ventilation rates were controlled using the chamber’s damper system (Accuvalve, Accutrol^®^ Systems, Monroe, CT) and validated by concentration decay studies [10]. Decay data were collected for 20 min following each simulation, to confirm the ventilation rates. Simulations 1 through 3 were conducted at low air-exchange rates (~0.78) and Simulations 4 through 6 were conducted at higher air-exchange rates (~2.05), representing residential conditions of window closed and open, respectively. Chamber study parameters are listed in Table 1.

**Table 1:** Test conditions for simulated floor cleaning conducted in exposure chamber

Test	Application rate (ml/min)	Area covered (m^2^/min)	Duration (min)	*Q* (m^3^/min)	Air changes per hour (h^−1^)	Location (height, m)
1	10	0.09	6	0.133	0.57	1.15
2	10	0.09	6	0.162	0.81	1.15
3	10	0.09	6	0.171	0.86	1.15
4	10	0.09	6	0.343	1.7	1.15
5	10	0.09	6	0.383	1.9	1.15
6	10	0.09	6	0.493	2.5	1.15

Tests 1– represent “Low” ventilation rates and tests 4–6 represent “high” ventilation rates

Time-varying airborne concentration of the floor cleaner’s volatile and semi-volatile components were measured with a portable Fourier Transform Infrared Spectrometer (FTIR) model DX 4040 (Gasmet^TM^, Helsinki, Finland). To ensure a high degree of measurement accuracy, the FTIR was challenged with a precision mixture of acetic acid using a FlexStream^TM^ automated permeation tube system fitted with a Trace Source™ permeation tube (Kin-Tek Analytical, Inc., Houston, TX). Precision concentrations of acetic acid were generated by varying the rate at which nitrogen, the dilution gas (source, location), passed over the permeation tube, from which a calibration curve was created.

### Modeling the time-varying generation rate

The Small Spill model [13] is a generation rate model that accounts for the contaminant mass applied at time *t* and evaporating at an exponentially decreasing rate. It was selected as the candidate model to measure the evaporation rate and to estimate the generation rate for this scenario in which each application of the cleaning mixture was treated as a small spill. Thus, for each simulation conducted in the chamber, there were a series of six sequential small spills at 1 min intervals. Similarly, for each simulation conducted in the field (test house), there were six sequential small spills. For the first application of the cleaning mixture to block 1, *G*_1_ (*t*_1_) is modeled as the first spill and is expressed by Equation 1.

1 $$G_1\left( t \right) = M_0ke^{ - kt}$$

where

*G*_1_(t) is the generation rate at time *t* (mg/min)

*M*_0_ is the mass applied at *t* = 0 (mg)

*k* is the unknown evaporation rate (min^−1^)

*t* is the time over which the first spill occurs, 0 < *t* < 1

After mopping block 1 for 1 min, the second aliquot of cleaning mixture is applied to block 2 and block 2 is mopped for 1 min, ending at *t* = 2 min. Now the airborne generation rate includes the evaporation from the most recent “spill” plus the residual contribution from the previous spill in block 1, located adjacent to block 2. This relationship is expressed by Equation 2:

2 $$G_2\left( t \right) = G_1\left( t \right) + M_0ke^{ - k \ast \left( {t\_2 - t\_1} \right)}$$

where

*G*_2_(*t*) is the generation rate at time *t* (mg/min)

1 < *t* < 2

Thus, the general form for time-varying generation rate at each time point (minute) reflecting the cumulative contribution of each previously mopped floor block is expressed as

3 $$G_n\left( t \right) = M_0ke^{ - kt}\left[ {\frac{{1 - e^{ - kn}}}{{1 - e^k}}} \right]$$

where

*G*_n_(*t*) is the time-varying generation rate (mg/min)

*n* is current application/spill number (*n* − 1) < *t* < *n*

In the above set of equations, the evaporation rate *k* is unknown. To solve for *k* and hence estimate *G*_n_(*t*), it is necessary to back-calculate, solving for these model inputs by using the concentration measurements, *C*, collected in the chamber. The WRM model was used for this purpose, as it provides a reasonable estimate of the average airborne concentration of a chemical emanating from a non-point source or multiple-point sources distributed throughout a room [13, 16]. The model and its assumptions are described elsewhere [13, 16] and will only be briefly described here. The time-varying equation is useful for estimating the concentration of a chemical evaporating into the room air from a non-point source such as the floor, as a function of time. The general equation for the WMR model as a function of time is expressed as:

4 $$C\left( t \right) = \hskip 1.5pt \frac{{G_n\left( t \right) + C_{in} \times Q}}{{Q + k_L \times V}}\left[ {1 - exp\left( { - \frac{{Q + k_L \times V}}{V} \times t} \right)} \right] \\ + C_{\left( 0 \right)}exp\left( { - \frac{{Q + k_L \times V}}{V} \times t} \right)$$

where

*C*(*t*) is the concentration at time, *t* (mg/m^3^)

*G*_n_(*t*) is the time-varying generation rate (mg/min)

*C*_in_ is the concentration in incoming air (mg/m^3^)

*C*_0_ is the initial concentration at time = 0

*Q* is the ventilation rate (m^3^/min)

*k*_L_ is the loss factor accounting for non-ventilatory losses due to various loss mechanisms such as sorption onto or into surfaces or particle settling (min^−1^)

*V* is the room volume (m^3^)

**Fig. 2:**
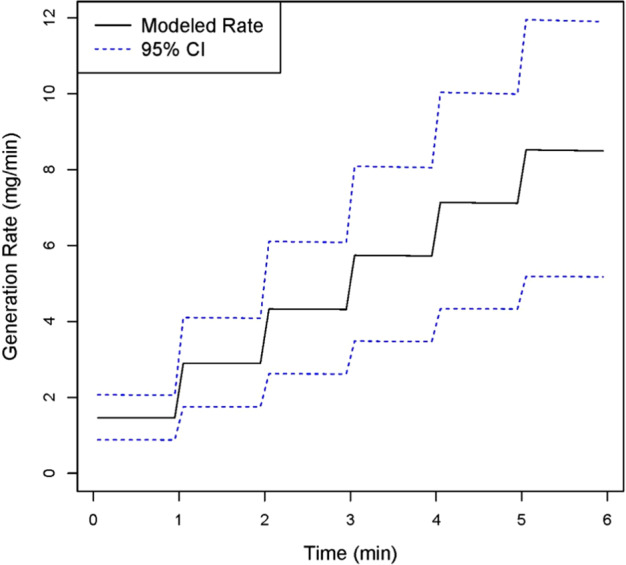
Time-varying generation rate estimated from chamber-generated values of *k*. A distribution of time-varying generation rates *G*_n_(*t*) was estimated by solving for *G*_n_(*t*) using the small spill model. *G*_n_(*t*) reflects the range of values of *k*, resulting from differences in measurement averaging time and change in mass of acetic acid applied across each 6 min simulation

One-minute time-weighted average (TWA) concentrations were calculated from the acetic acid measurements. Knowing all other model inputs and using the expression for the generation from Equation 3, the time-varying generation rate was estimated by first solving for *k* using ordinary least squares (OLS) with the six TWA concentrations in each test. Using OLS, the value of *k* that minimized the squared sum of differences between the measured and modeled acetic acid concentration was found. This best fit value of *k* was then used to estimate a time-varying *G* for each test producing six estimates for *G*(*t*) corresponding to the six tests (Fig. 2). Model input values for both models are shown in Table 2. To evaluate the influence of averaging time on *k*, a series of values of *k* were generated for exposure averaging times ranging from 30 s TWAs to 6 min TWAs (Table 3).

**Table 2:** Small spill and WMR model inputs used to solve for *k* and *G*_n_(*t*)

Parameter	Value
Generation rate *G*(*t*), mg/min.	(Estimated after solving for *k*)
Contaminant mass *M*, mg	420
Time *t*, minutes	1 to 6
Evaporation rate *k*, min^−1^	0.002–5 (Solved for)
Ventilation rate *Q*, m^3^/min	
Simulation 1	0.133
2	0.162
3	0.171
4	0.343
5	0.383
6	0.493
Room volume *V*, m^3^	11.9
Concentration in incoming air *C*_in_	0
Initial concentration, *C*_0_	0
Loss factor kL, min^−1^	0

**Table 3:** Evaluating the influence of averaging times on *k*

	ACH				
Averaging Time	0.57	0.82	0.86	1.7	2.5
			k (min^−1^)		
30 s	0.0016	0.0020	0.0017	0.0017	0.0017
1 min	0.0027	0.0030	0.0029	0.0029	0.0029
2 min	0.0040	0.0050	0.0044	0.0040	0.0060
3 min	0.0045	0.0045	0.0050	0.0054	0.0050
6 min	0.0040	0.0054	0.0048	0.0050	0.0050

### Field study

A field study was conducted in a residential setting to evaluate the portability of *G*_n_*t*. This setting was a single family home, built in 1911. It is ~1140 square feet and has two bedrooms and one bathroom located on the second floor of the home. The house has forced-air furnace and air conditioning (Model CKL 24-1 F, Goodman Manufacturing Company, Houston, TX). Simulations were conducted in an all-purpose room with a hardwood floor located on the first floor and in a bathroom with a ceramic tile floor located on the second floor. One Air King adjustable-height three-speed was set on the lowest setting, directed away from the spill to create sufficient air turbulence to ensure good mixing. Measurements of the airborne acetic acid concentrations were collected in the personal breathing zone using the portable FTIR, following the same approach as in the chamber studies. Three simulations in the all-purpose room were conducted initially with the exterior door closed (Room 1) and then repeated three times with the door open (Room 2). The application rate and mass loading were consistent with the conditions applied in the chamber tests, and were controlled to ensure consistent application across a consistent surface area. Floor area for the field study was consistent with the chamber study, resulting in a six block 0.56 m^2^ (6 ft^2^) test area. A central air-conditioning system was set to maintain room temperatures at 21 °F. Ventilation rates were measured by concentration mass decay (Table 4). The range of air exchange rates in the field study were similar to those used in the chamber study, making *k* portable for these field conditions. An oscillating floor fan was turned on to the lowest setting and positioned to face the back wall in the all-purpose room during the simulations, to ensure reasonably good mixing. In the bathroom (Room 3), good mixing was assumed based on the constant influx of cool air from the air condition system but no mechanical interventions were applied to induce mixing. Three replicate simulations were conducted with the bathroom door closed.

**Table 4:** Field study model inputs used to predict airborne acetic acid concentrations

Room	Floor substrate	Volume (m^3^)	Ventilation rate (m^3^/min)	Air changes per hour
All purpose^1^	Hardwood	24.46	0.5	1.22
All purpose^2^	Hardwood	24.46	0.67	1.60
Bathroom	Ceramic tile	8.9	0.505	3.4

### Modeling residential acetic acid concentrations

Acetic acid concentrations were modeled using the WMR model, incorporating the chamber-generated values for *k* and *G*_n_(*t*). There are several factors that suggest the WMR model is a good model candidate for this scenario. First, all three rooms were relatively small with the room air and any acetic acid in it relatively well mixed, especially with the use of the fan in this study to induce good mixing. Second, dispersive, non-point emission sources are generated by the series of small spills; third, the small room volumes would not require much time for the concentration mass to be relatively uniformly distributed. In scenarios where the surface cleaned is a counter top rather than a floor, the close proximity of the surface to the breathing zone may result in sufficient spatial difference in exposure intensity to favor the Near Field Far Field model [14–23].

Monte-Carlo simulations were used to model residential acetic acid concentrations and generation rates. The simulations were performed using R version 3.5.1 [24]. A total of 1000 simulations were run, accounting for the uncertainty of both the evaporation rate and the ventilation rate. The evaporation rate, *k*, was modeled as a uniform random variable with bounds at the minimum and maximum estimated values (from the chamber study). The ventilation rate, *Q*, was modeled as a normal truncated random variable, constrained by zero using sample means and SDs from measurements taken in each of the three rooms. The time steps modeled represented every 3 s from the initial spill, excluding exact minute values to conform to the strict inequalities in Equation 3.

## Results and discussion

### Estimating the evaporation rate and time-varying generation rates

**Fig. 3:**
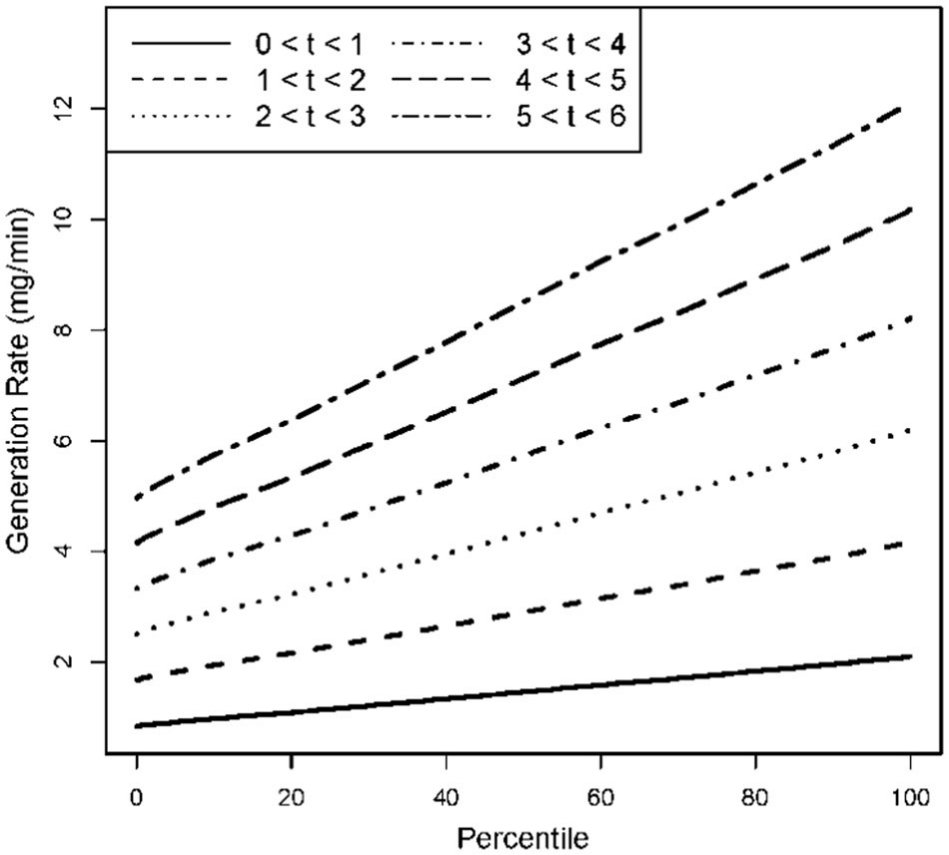
Cumulative plot of time-varying generation rates. After solving for the evaporation rate, *k*, accounting for differences associated with measurement averaging time, a range of values for *k* was generated. By incorporating this range of values of *k*, a distribution of time-varying generation rates was generated for each “small spill” over a 6 min period

Evaporation rates were estimated from each chamber study test, applying the small spill and WMR models to solve for the best estimate of *k*. Estimates of *k* were relatively consistent across air exchange rates. For 1 min averaging times, *k* ranged from 0.0027 to 0.0035 min^−1^, except simulation 5. Measurement collection during this simulation was inadvertently delayed until after the simulation started, so data from this simulation was excluded from the study. *G*_n_(*t*) was thus calculated for simulations 1–4 and 6, for each of the five simulations generated from the six “small spills.” The impact of averaging time on *k* was evaluated by solving for *k* using averaging times of 30 s (the instrument averaging time), 1, 2, 3, and 6 min. Estimates of *k* remained constant across all five simulations for each averaging period but increased with increasing averaging time. The 30 s averaging time corresponded to a value of *k* = 0.0016. For *k* estimated from 3 and 6 min averaging times, *k* = 0.005 (Table 3). Thus, distribution of values of *k* encompassed all of these values. *G*_n_(*t*) was then estimated for each 1 min interval according to equations 1–3. The cumulative distributions of mean values for the simulated generation rates *G*_n_(*t*) are presented in Fig. 3.

### Evaluating the generation rate

**Fig. 4:**
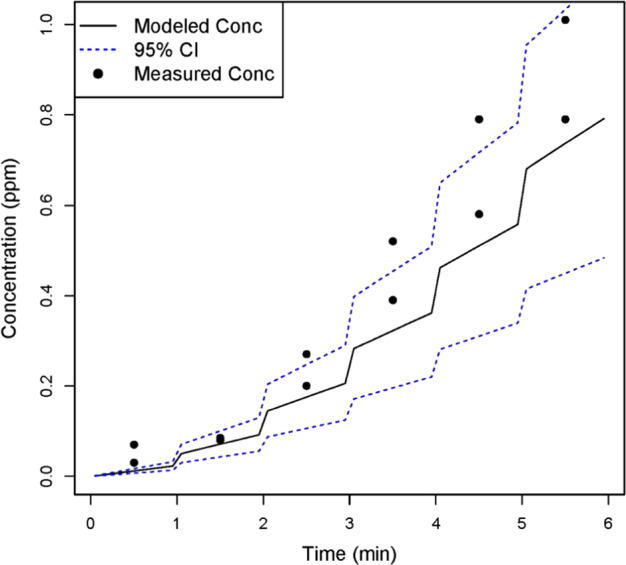
Measured and modeled (WMR model) acetic acid concentrations in Room 1. Measured airborne acetic acid concentrations and modeled airborne concentrations, based on *G*_n_(*t*), were compared for Room 1. Most of the measured concentrations were within the 95% confidence interval of the modeled estimates, suggesting that the time-varying generation rate provides reasonably accurate exposure estimates

**Fig. 5:**
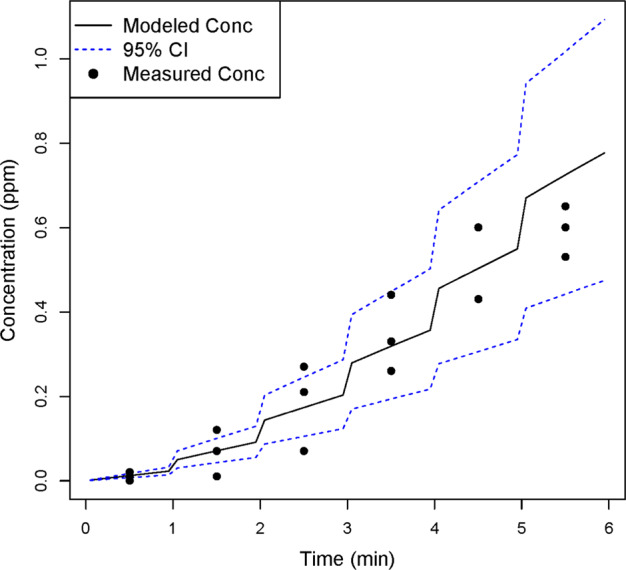
Measured and modeled (WMR model) acetic acid concentrations in Room 2. Measured airborne acetic acid concentrations and modeled airborne concentrations, based on *G*_n_(*t*) were compared for Room 2. The majority of the measured concentrations were within the 95% confidence interval of the modeled estimates, suggesting that the time-varying generation rate provides reasonably accurate exposure estimates

**Fig. 6:**
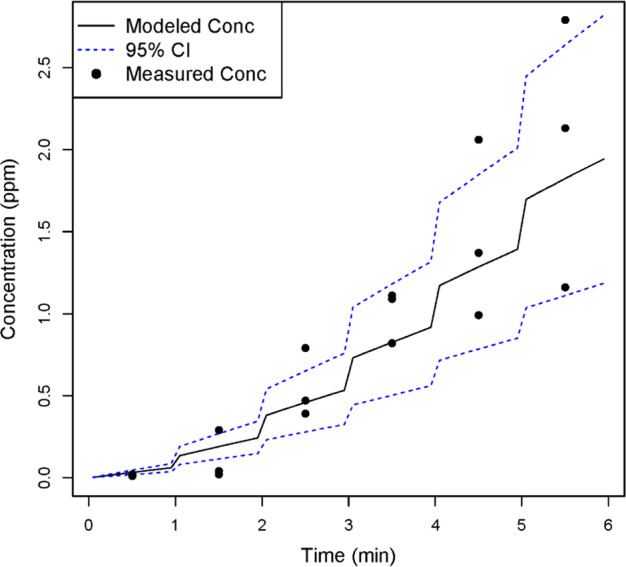
Measured and modeled (WMR model) acetic acid concentrations in Room 3. Measured airborne acetic acid concentrations and modeled airborne concentrations, based on *G*_n_(*t*) were compared for Room 3. The majority of the measured concentrations were within the 95% confidence interval of the modeled estimates, suggesting that the time-varying generation rate provides reasonably accurate exposure estimates

The portability of the evaporation and generation rates were evaluated by modeling acetic acid concentrations for three field scenarios: a residential all-purpose room with the doors closed (Room 1), the same room with the exterior door open (Room 2), and a bathroom (Room 3). The time-varying modeled and measured acetic acid concentration estimates are shown in Figs. 4–6. The measured concentrations in Room 1 fall within or just slightly above the 95% confidence interval (CI) of the WMR modeled concentrations. Similarly, for Rooms 2 and 3, the 95% CI WMR modeled time-varying concentrations encompass most of the measured concentrations.

TWA-measured and modeled acetic acid concentrations were also compared, to further evaluate the portability of the time-varying generation rate beyond the chamber environment. Specifically, we compared mean modeled concentrations that were calculated from a distribution of modeled TWA values, to test the usefulness of the time-varying generation rate. The TWA-measured acetic acid concentrations were calculated from field measurements. Results are shown in Table 5. Modeled TWA concentrations using the WMR model aligned closely with the measured TWA concentrations, albeit underestimating the concentration in Room 1 (74% of measured TWA) and Room 3 (90% of measured TWA). For Room 2, the predicted TWA exposure exceeded the measured TWA (107%). Furthermore, these results suggest that the time-varying generation rate are useful in predicting average airborne concentrations from surface cleaning with aqueous acetic acid mixtures, well beyond the chamber environment.

**Table 5:** Time-weighted average-measured and modeled acetic acid concentrations

Time -weighted average concentration (p.p.m.)		
	Measured	WMR
Room 1	0.413 (0.334)	0.305 (0.272)
Room 2	0.281 (0.229)	0.301 (0.267)
Room 3	0.864 (0.822)	0.777 (0.681)

Showing mean and (SD) concentrations averaged over 6 min (*n* = 3)

*WMR* Well Mixed Room model

This study demonstrates the value of determining reasonably accurate generation rates to develop accurate exposure scenarios. In the absence of this critical model input, the use of reasonable worst-case model inputs significantly overestimates the emission rate, especially when estimating emissions from chemical mixtures. Previous emission rate estimates [1] for an 8% acetic acid floor cleaning solution resulted in a predicted concentration of 4470 mg/m^3^. Assuming a 4% solution would have resulted in one-half of this concentration so an estimated 2,235 mg/m^3^. These values, born of worst-case estimates because of a lack of a good experimental value for *G*, are almost three orders of magnitude above the carefully measured or refine-modeled estimates of this work.

The scenario simulated in the chamber was designed to represent reasonable worst case, with a 4% acetic acid mixture applied to the floor at a conservative rate and mopping the area for 1 min before moving to the next area. As mentioned above, the *G*_n_(*t*) presented here represents a significant refinement relative to previously estimated generation rate values for this mixture using worst-case assumptions (Maier et al. [1]). More accurate model inputs such as the *G*_n_(*t*) evaluated here can be expected to yield more accurate exposure assessments of consumer products, facilitating more efficient and, importantly, effective risk assessments. Although the small spill model could have also been used to back-calculate for *k* and *G*, and evaluate the portability of *G*_n_(*t*), the results of the WMR model, which is computationally less laborious, show that this model provided reasonable agreement between measured and modeled estimates.

The field study, incorporated to evaluate how well-modeled concentrations based on these rates reflect real-world concentrations, suggests the empirically estimated evaporation and generation rates are indeed portable. That is, when incorporated into the WMR model, the 95% CI of the predicted acetic acid concentrations encompassed most of the measured concentrations. The evaporation rate will be useful for estimating generation rates for other, similar indoor surface-cleaning scenarios, even when the application time and impacted area differ. Importantly, this analysis has broad application to a wide range of scenarios benefitting from refinement of screening level exposure estimates of volatile or semi-volatile chemicals.

There are several limitations to this study. The concentration of acetic acid was deemed an upper-bound concentration expected in a consumer product and the delivery using a pipette was intended to ensure consistent delivery throughout the study but may underestimate real-world variability. The evaporation rate *k* and generation rates based on *k* would be influenced by temperature of the liquid and air temperature above it, humidity, airflow over the pool, and thickness and surface area of the liquid. These factors were held constant in this study due to scope and resource limitations. Future studies focused on quantifying these influences would be helpful. The generation rates estimated from these studies are conservative to the degree that the formulation strength and applied mass are truly upper-bound concentrations and loads. The concentrations of airborne acetic acid were measured in one location, with the inlet positioned at breathing zone height. An implicit WMR model assumption is that the contaminant in the room air is well mixed. Another assumption is that these measurements represent personal exposures with a reasonable degree of accuracy. Rigorously validating these assumptions would require an additional investment of resources.

## Conclusions

The study design presented here was useful for estimating a reasonably accurate time-varying generation rate for acetic acid evaporating from a cleaning product formulation used to mop floors. This generation rate can also be applied to a broader set of scenarios involving surface cleaning using aqueous acetic acid mixtures, addressing the need to refine exposure assessments based on screening-level assumptions. This chamber and field study approach could be used to estimate generation rates for other mixtures. It will be particularly useful when refined exposure assessments involving chemical mixtures are needed but generation rates are not publicly available in the peer-reviewed literature. Finally, the importance of considering model assumptions in selecting the best model for predicting exposures was evident in the agreement between measured and predicted concentrations using the WMR model.

## Data Availability

The modeling code is included as a Supplementary File.
